# Fissure of the Anus

**Published:** 1874-10

**Authors:** Samuel D. Turney


Fissure of the Anus—*By Samuel D. Turney, M.D.*—This very painful disease has heretofore been relegated to the department of operative surgery.

Some three years since, in seeking for a palliative for a patient who deferred an operation to a more convenient season, I prescribed a suppository of iodoform. To my surprise, it effected a radical cure.

Constipation should be overcome by a laxative. I use a pill of—

| | |
|---|---|
| Ex. Nux Vomica, . . . . . | $\frac{1}{2}$ gr. |
| Ex. Belladonna, . . . . . . | $\frac{1}{3}$ gr. |
| Ex. Aloes, . . . . . . . . | 1 gr. |
| Ipecac, . . . . . . . . . | 1 gr. |

To be taken at bed time.

Immediately after the bowels move, use a suppository of—

| | |
|---|---|
| Iodoform, . . . . . . . . | 5 grs. |
| Butter of Cacao, . . . . . . | q. s. |

I hope this treatment may be as successful in the hands of others as it has been in my own.—*The Clinic.*

